# Erbin accelerates TFEB-mediated lysosome biogenesis and autophagy and alleviates sepsis-induced inflammatory responses and organ injuries

**DOI:** 10.1186/s12967-023-04796-y

**Published:** 2023-12-17

**Authors:** Qing Fang, Guoqing Jing, Ying Zhang, Hongyu Wang, Huan Luo, Yun Xia, Qiyan Jin, Yuping Liu, Jing Zuo, Cheng Yang, Xiaodong Zhang, Shi Liu, Xiaojing Wu, Xuemin Song

**Affiliations:** 1 The Research Centre of Anesthesiology and Critical Care Medicine, Zhongnan Hospital of Wuhan University, Wuchang, 169 Donghu Road, Wuhan, 430071 Hubei Province China; 2 Department of Anesthesiology, Renmin Hospital of Wuhan University, Wuchang, 238 Liberation Road, Wuhan, 430060 Hubei Province China; 3 College of Life Sciences at, Wuhan University, Wuchang, 299 Bayi Road, Wuhan, 430072 Hubei Province China

**Keywords:** Erbin, Autophagy, Lysosome, TFEB, Sepsis

## Abstract

Mounting attention has been focused on defects of the autophagy-lysosomal pathway in sepsis, however, the precise mechanisms governing the autophagy-lysosomal process in sepsis are poorly known. We have previously reported that *Erbin* deficiency aggravated the inflammatory response and organ injuries caused by sepsis. In the present study, we found that *Erbin* knockout impaired the autophagy process in both muramyl dipeptide (MDP)-induced bone marrow-derived macrophages (BMDMs) and sepsis mouse liver and lung, as detected by the accumulation of LC3-II and SQSTM1/p62, and autophagosomes. Pretreatment with autophagy inhibitor chloroquine (CQ) further aggravated inflammatory response and organ injuries in vivo and in vitro sepsis model. We also observed that the impaired lysosomal function mediated autophagic blockade, as detected by the decreased expression of ATP6V, cathepsin B (CTSB) and LAMP2 protein. Immunoprecipitation revealed that the C-terminal of Erbin (aa 391–964) interacts with the N-terminal of transcription factor EB (TFEB) (aa 1–247), and affects the stability of TFEB-14-3-3 and TFEB-PPP3CB complexes and the phosphorylation status of TFEB, thereby promote the nucleus translocation of TFEB and the TFEB target genes transcription. Thus, our study suggested that *Erbin* alleviated sepsis-induced inflammatory responses and organ injuries by rescuing dysfunction of the autophagy-lysosomal pathway through TFEB-14-3-3 and TFEB-PPP3CB pathway.

## Introduction

Autophagy is a dynamic lysosome-dependent conserved catabolic process responsible for processing damaged organelles, metabolizing macromolecules and intracellular microbes to maintain cellular homeostasis and playing an essential role in cancer, neurodegenerative, metabolic, and inflammatory disorders [1, 2]. Autophagy could coordinate macrophage polarization, inflammasome activation and release of inflammatory cytokines, and tissue injury during sepsis [3]. Classical autophagy/macroautophagy initiates the generation of a double-membrane vesicle called the autophagosome. Then the outer membrane of the autophagosome fuses with the lysosomal membrane to form an autolysosome, in which the cytosolic materials are degraded by the cathepsins and hydrolytic enzymes [4]. Any perturbed step might result in the dysfunction of autophagy and human diseases.

The current new view is that lysosomes are no longer just static organelles that process and recycle cellular waste but highly dynamic structures that mediate the adaptation of cell metabolism to the environment [5, 6]. Lysosomes are acidic organelles containing more than 50 different hydrolases, and proper function of these enzymes requires a low pH (4.5–5.0) within the lysosome, maintained by empty Vacuolar-type proton pumping ATPase (V-ATPase), providing a suitable condition for lysosomal degradation of its contents [7]. In the course of autophagy, the process of degradation and recycling strictly depends on the lysosomal function, as the contents in the autophagosomes are eventually degraded by lysosomes via autophagosome-lysosome fusion [8–10]. Xia et al. [11] found that the V-ATPase 6V0D2 gene defect impaired the acidic environment of macrophage lysosome, led to the accumulation of autophagy proteins LC3-II and p62 in macrophages and the blockage of autophagic flux, and aggravated the mitochondrial damage and inflammatory response. In addition, they found that V-ATPase 6V0D2 gene knockout also aggravated Dextran sulfate sodium (DSS) -induced colitis in mice and Salmonella typhi-induced death in animals.

Lysosomes can mediate signaling pathways and transcriptional programs that sense the metabolic state of cells by regulating lysosomal biogenesis and autophagy, most of which depend on transcription factor EB (TFEB) [8]. TFEB is one of the MiT-TFE family members, which can combine a palindromic DNA sequence located in the proximal promoter of target genes and regulate the expression of lysosomal and autophagy-related genes [12]. Normally, phosphorylated TFEB binds to the molecular chaperone YWHA/14-3-3 in the cytoplasm. Under stress conditions, such as starvation or infection, cytoplasmic TFEB is rapidly dephosphorylated. The dephosphorylated TFEB dissociates from molecular companion protein 14-3-3 and transfers from cytoplasmic into intracellular nuclei, activating lysosomal and autophagy-related gene expression. Meanwhile, the balance between phosphorylation and dephosphorylation of TFEB by PPP3CB determines cytoplasmic and nucleus distribution [13–15].

Erbin, a newly identified PDZ protein that acts as an adaptor for the ERBB2/HER2 in epithelia, contains 16 leucine-rich repeats (LRRs) in its amino terminus and a PDZ domain at its carboxy terminus [16]. Erbin was discovered as a regulator of inflammation response due to its ability to bind specifically to intracellular receptor nucleotide-binding oligomerization domain-containing protein 2 (NOD2), which is one of the pattern recognition molecules (PRMs), detecting peptidoglycan (PGN) through the recognition of muramyl dipeptide (MDP) common to bacteria, and then up-regulation of pro-inflammatory cytokines [17, 18]. The present study showed that Erbin alleviated the inflammatory response and organ injuries caused by sepsis by promoting lysosomal biogenesis and autophagy, bridging the immune system and the core autophagosomal machinery. Mechanically, Erbin interacts with TFEB and affects the stability of TFEB-14-3-3 and TFEB-PPP3CB complexes, promoting the activation of TFEB and the nucleus translocation, thus regulating the lysosomal biogenesis. Our results revealed a novel mechanism of autophagy activation regulated by Erbin protein, leading to the suppression of inflammatory response and avoiding organ injuries of sepsis.

## Materials and methods

### Materials 

MDP was purchased from Sigma-Aldrich (A9519). Lipofectamine-3000 (L3000015) was purchased from Invitrogen Corporation. Rapamycin (RAPA) was purchased from Solarbio (R8140). Chloroquine (CQ) and M-CSF were purchased from MedChemExpress (HY-W031727). Lysotracker Red (C11046) and Hoechst 33342 (C1022) were purchased from Beyotime Biotechnology. Unless specified otherwise, all biochemical reagents were purchased from Sigma-Aldrich. Mammalian expression plasmids for HA-tagged Erbin, Myc-tagged TFEB, and its mutants were constructed by standard molecular biology techniques. To verify constructs and the specificity of antibodies, all constructs were transfected into 293T cells, and expression was analyzed using Western blot. All constructs were confirmed by DNA sequencing.

The antibodies used for western blot were anti-Erbin (Novus Biologicals, NBP2-56104), anti-LC3-I/II (Cell Signaling Technology (CST), 4108s), anti-SQSTM1/P62 (CST, 8025S), anti-Beclin1 (CST, 3495), anti-ATG5 (CST, 12994s), anti-ATG7 (CST, 8558s), anti-Cathepsin B (CST, 31718), anti-ATP6V1A (ABclonal, A14706), anti-ATP6V1B2 (ABclonal, A16770), anti-LAMP2 (Santa Cruz, G1619), anti-β-actin (CST, 4970s), anti-FLAG (Sigma, F1804), anti-GFP (Abcam, ab290), anti-PPP3CB (Abcam, ab58161), anti-pan 14-3-3 (Santa Cruz Biotechnology, sc-133232), anti-TFEB (CST, 4240), anti-phospho (Ser)-14-3-3 binding motif (CST, 9601), anti-TFEB (Thermo Pierce, PA1-31552), anti-phospho-TFEB (Ser211) (E9S8N) (CST, 37681). HRP conjugated secondary antibodies, Pierce(R) Goat Anti-Rabbit IgG, (H&L) (#31460) and Pierce(R) Goat Anti-Mouse IgG, (H&L) (#31430), were purchased from Thermo.

### Cells culture and treatment

Bone marrow-derived macrophages (BMDMs) were collected from the femurs of Erbin^fl/fl^ and Erbin^fl/fl−Lyz2−Cre^ mice, and cells were simultaneously lysed in 1 × ACK buffer. Then, the BMDMs were cultured in DMEM (Hyclone) supplemented with 10% fetal bovine serum (HyClone, Logan, UT, USA), 100 U/ml of penicillin and 100 μg/ml of streptomycin, and M-CSF (20 ng/ml) in a humidified atmosphere containing 5% CO_2_ at 37 °C. 7 days later, the supernatant was removed, and the cells were harvested via trypsin digestion and cultured for further experiments. Cells were treated with MDP (10 μg/ml) for 6 h with or without various agonists or inhibitors according to the experimental design. Cells were collected and homogenized immediately to extract RNA or protein or fixed neutral-buffed formalin for staining. Cells were stained with LysoTracker Red to detect the acidic microenvironment of the lysosome. Confocal images were obtained using an FV10i FLUOVIEW Confocal Microscope (Olympus, Tokyo, Japan). The human monocytic cell line THP-1 was a gift from Pro. Ying Zhu of Wuhan University. THP-1 cells were differentiated to macrophages with 60 nM TPA for 12–14 h, and cells were cultured for 24 h without TPA. THP-1 cells were cultured in RPMI-1640 medium (Sigma, USA) supplemented with 10% heat-inactivated FBS (Sigma, USA). HeLa and HEK293T cells were from the American Type Culture Collection. The HIV molecular clones pNL-4-3∆Env or control lentiviral vector were transfected in 293T cells with vesicular stomatitis virus-G envelope. After 24 h of transfection, the supernatant was collected, filtered, and normalized for viral budding by ELISA (ZeptoMetrix).

### Animals

*Erbin* conditional knockout mice and Lyz2-Cre were obtained from Cyagen (Guangzhou) Biosciences. Erbin^fl/fl/Lyz2−Cre^ mice were obtained by crossing the Erbin flox mice with Lyz2-Cre mice. *Erbin* whole gene knockout mice in the C57BL/6 background were generated by Wuhan Xianran Biological Technology Co. LTD. Erbin^−/−^ mice were bred with C57BL/6 mice to obtain Erbin^±^ heterozygotes. Erbin^−/−^ mice and littermate control obtained from heterozygote crosses were used for all experiments. All mice were male, eight weeks old at the time of use. Mice were maintained in specific pathogen-free conditions under a 12-h/12-h light/dark cycle and allowed ad libitum access to water and a standard laboratory diet but deprived of food just before cecal ligation and puncture (CLP). Control mice were also deprived of food in the same manner. All procedures and treatments were conducted by the ethical regulations set by the Animal Experimentation Committee of Wuhan University (WQ20210298).

### Cecal ligation and puncture

Mice were anesthetized with sevoflurane. A 1- to 2-cm ventral midline incision was performed, and the cecum was isolated. The feces at the upper end of the cecum were gently squeezed to fill the end of the cecum. Ligation was performed at the midpoint of the cecal valve and cecum with sterile No.4 silk thread, and a 21G sterile needle was used to puncture the cecum at the midpoint of the ligation site and the top of the cecum. Gently squeeze the cecum, extrude a little content to ensure the perforation is unobstructed, wipe the extruded content, then push the cecum back to the abdominal cavity, close the abdominal cavity, and layer by layer suture. Postoperative fluid resuscitation was performed with normal saline (37 °C, 5 ml/100 g) and buprenorphine (0.05 mg/kg) for analgesia. Sham-operated animals received the same surgical procedures without ligation and puncture. Animals had free access to food and water postoperatively. To inhibit autophagosome-lysosome formation during sepsis, chloroquine (CQ) (60 mg/kg) was pre-injected intraperitoneally. Mice were subjected to CLP and killed at predetermined times to examine time-dependent effects. The serum was collected for biochemical analysis. The liver and lungs were collected and frozen immediately in liquid nitrogen for western blot or fixed in neutral-buffered formalin for histochemical examination.

### Bronchoalveolar lavage fluid (BALF) collection and inflammatory cell counting and protein concentration determination 

Mice were euthanized at the experimental time, and the lungs were lavaged with 1 ml PBS through the tracheal cannula to obtain BALF. The BALF was centrifuged at 800 × for 15 min at 4 °C, and cell pellets were resuspended in PBS. The total number of inflammatory cells in the BALF was determined by counting the cells with a hemocytometer. The polymorphonuclear neutrophils (PMNs) cells were fixed and stained using Wright Stain solution. Then they were classified by a laboratory technologist blinded to the experimental design to determine the percentage of neutrophils. The total protein concentration in the supernatants was determined by the BCA method.

### Western blot and immunoprecipitation assay

Immunoprecipitation was performed by lysing cells in IP buffer (50 mM Tris-HCI 8.0, 5 mM NaCl, 1 mM EDTA, 0.1% NP40). Cell lysates containing 1 mg protein of each treatment were incubated at 4 °C with primary antibody for 4 h before Dynabeads protein G (Thermo Scientific) was added to the samples. After further incubation for 4 h at 4 °C, beads were washed three times in ice-cold lysis buffer, and the immunoprecipitated complexes were eluted by boiling for 5 min in 1.5 × SDS buffer. Lastly, the eluted immunoprecipitated complexes were resolved for immunoblotting analysis.

### Immunofluorescence

BMDMs were seeded at 1 × 10^5^ cells on the coverslips and rested overnight for proper attachment. After treatment, cells were washed twice with sterile PBS, fixed with 4% ice-cold paraformaldehyde (PFA), then permeabilized with 0.25% Triton X-100 and blocked in 3% bovine serum albumin (BSA). Incubated with primary antibody overnight at 4 °C. The next day, coverslips were incubated with FICT-conjugated secondary antibody for 1 h at 37 °C. The coverslips were stained with DAPI and photographed under fluorescence microscopy.

### Transmitted electron microscopy

BMDMs were fixed in a fixative buffer containing 2.5% buffered glutaraldehyde and 2% paraformaldehyde in 0.1 M of phosphate-buffered solution at 4 ℃ for 1 h, then fixed with 1% osmic acid for 1 h, washed with distilled water, and embedded in Epon-812. Samples were cut into ultrathin section (70 nm) using an LKB-V ultramicrotome (Bromma, Sweden) and stained with 0.2% lead citrate and 2% uranyl acetate. The sections were examined with a Hitachi H-600 transmission electron microscope (Hitachi, Tokyo, Japan).

### Isolation of total RNA and qPCR

Total RNA was extracted using the TRIzol reagent (Invitrogen) according to the manufacturer’s instructions. Real-time quantitative PCR (qPCR) analysis was performed using the Roche LC480 and SYBR RT-PCR kits (DBI Bioscience, Ludwigshafen, Rhineland-Palatinate, Germany) according to the manufacturer’s instructions. The data were normalized according to the level of β-actin expression in each sample.

### Co-immunoprecipitation (Co-IP)

Cells were collected and lysed in IP-lysis buffer (50 mM Tris–HCl, 150 mM NaCl, 1% Triton X-100, 1 mM EDTA, 10% glycerol, and protease inhibitor cocktail, pH7.4). Supernatants were collected by centrifugation (15,000 g, 15 min, 4 °C) and were pre-cleared with 30 μl protein G-conjugated agarose (GE Healthcare Life Sciences) followed by centrifugation (2000 g, 2 min, 4 °C). The pre-cleared supernatants were incubated with the indicated antibodies (1 μg/ml) for 3 h or overnight at 4 °C, followed by immunoprecipitation with 30 μl protein G-conjugated agarose for 2 h at 4 °C. The precipitates were washed 5–7 times with IP-wash buffer (50 mM Tris–Cl, 300 mM NaCl, 1% Triton X-100, 1 mM EDTA, pH 7.4), and bound proteins were separated by SDS-PAGE with subsequent immunoblotting analysis.

### Western blotting

Total proteins were extracted by RIPA buffer (Beyotime Biotechnology, P0013E) containing complete protease inhibitors (Roche Applied Sciences). The protein concentration was quantified using the Pierce^®^ BCA Protein Assay Kit (Pierce, 23225). Samples containing the same amounts of proteins were then separated by sodium dodecyl sulfate–polyacrylamide gel electrophoresis in 8–15% acrylamide gels and transferred to polyvinylidene difluoride (PVDF, Millipore, IPVH00010) membranes. The membranes were blocked in 5% non-fat milk and incubated with indicated primary antibodies at 4 ℃ overnight. After incubating with horseradish peroxidase-conjugated secondary antibody, the bands were visualized by the ECL detection system (Millipore).

### Nuclear extraction

Cells were incubated in serum-free media for 24 h, washed twice with cold PBS, and scraped into 1 ml cold PBS. Cells were harvested by centrifugation (15 s) and incubated in two packed cell volumes of buffer A (10 mM HEPES, pH 8, 0.5% Nonidet P-40, 1.5 mM MgCl_2_, 10 mM KCl, 0.5 mM DTT, and 200 mM sucrose) for 5 min at 4 °C with the flipping of the tube. The crude nuclei were collected by centrifugation (30 s); pellets were rinsed with buffer A, resuspended in one packed cell volume of buffer B (20 mM HEPES, pH 7.9, 1.5 mM MgCl_2_, 420 mM NaCl, 0.2 mM EDTA, and 1.0 mM DTT), and incubated on a shaking platform for 30 min at 4 °C. Nuclei were centrifuged (5 min), and supernatants were diluted 1:1 with buffer C (20 mM HEPES, pH 7.9, 100 mM KCl, 0.2 mM EDTA, 20% glycerol, and 1 mM DTT). Cocktail protease inhibitor tablets were added to each type of buffer. Nuclear extracts were snap-frozen in liquid nitrogen and stored at − 70˚C until use.

### Protein purification

For in vitro pull-down assays, Erbin was cloned into the expression vector pGEX-4T-1 (Amersham Pharmacia, Buckinghamshire, UK) and was expressed in Rosetta (DE3) pLys (Novagen) *E. coli* cells. The recombinant proteins were purified on glutathione-sepharose bead (Pharmacia, Buckinghamshire, UK) columns to obtain relatively pure GST-Erbin. His-ATG16L, and His-TFEB were induced in the same manner and purified on the Ni–NTA columns (Qiagen, Germany). For the in vitro pull-down assays, GST-Erbin, and His-TFEB were incubated together in various combinations. After a short incubation, the reaction systems were immunoprecipitated using agarose-immobilized indicated antibodies or protein A/G agarose beads and were then analyzed by western blotting.

### Histological analysis

The liver and lung tissues were harvested to observe morphologic alterations at 6 h, 24 h, and 48 h after CLP. The lung’s right middle lobe and the liver’s right lobe were excised, washed, and fixed with 4% (v/v) paraformaldehyde for 24 h at 4 °C. The liver and lung tissues were embedded in paraffin, sectioned at 4 μm thickness, dewaxed and rehydrated, and stained with hematoxylin and eosin (H&E) solution (hematoxylin, MHS16; eosin, HT110132; Sigma-Aldrich, USA) to estimate pathological damages. The stained slides were then observed with a light microscope, and the digital micrographs were taken for analysis. Histologic changes were evaluated by a pathologist blinded to the experiment. The histological score of the lungs was calculated according to our study [29].

### Serum analysis

The blood was collected from the abdominal aorta, and the serum was obtained following centrifugation (1000 × g for 10 min). The levels of TNF-α, IL-6, IL-1β, HMGB1, and IL-10 in serum were determined using enzyme-linked immunosorbent assay (ELISA) kits according to the manufacturer's instructions (R&D Systems, Minneapolis, MN, USA). The absorbance was measured at 450 nm using an ELISA reader (BioTek Instruments, Inc., USA).

### Statistical analysis

Data were expressed as mean ± SEM. Statistical significance was analyzed with one-way analysis of variance (ANOVA) followed by post hoc (Bonferroni t); *P* values < 0.05 were considered significant. We used SPSS 23.0 to perform the statistical analysis.

## Results

### Erbin plays an anti-inflammatory protective role in polymicrobial sepsis animals and MDP-treated BMDMs

**Fig. 1:**
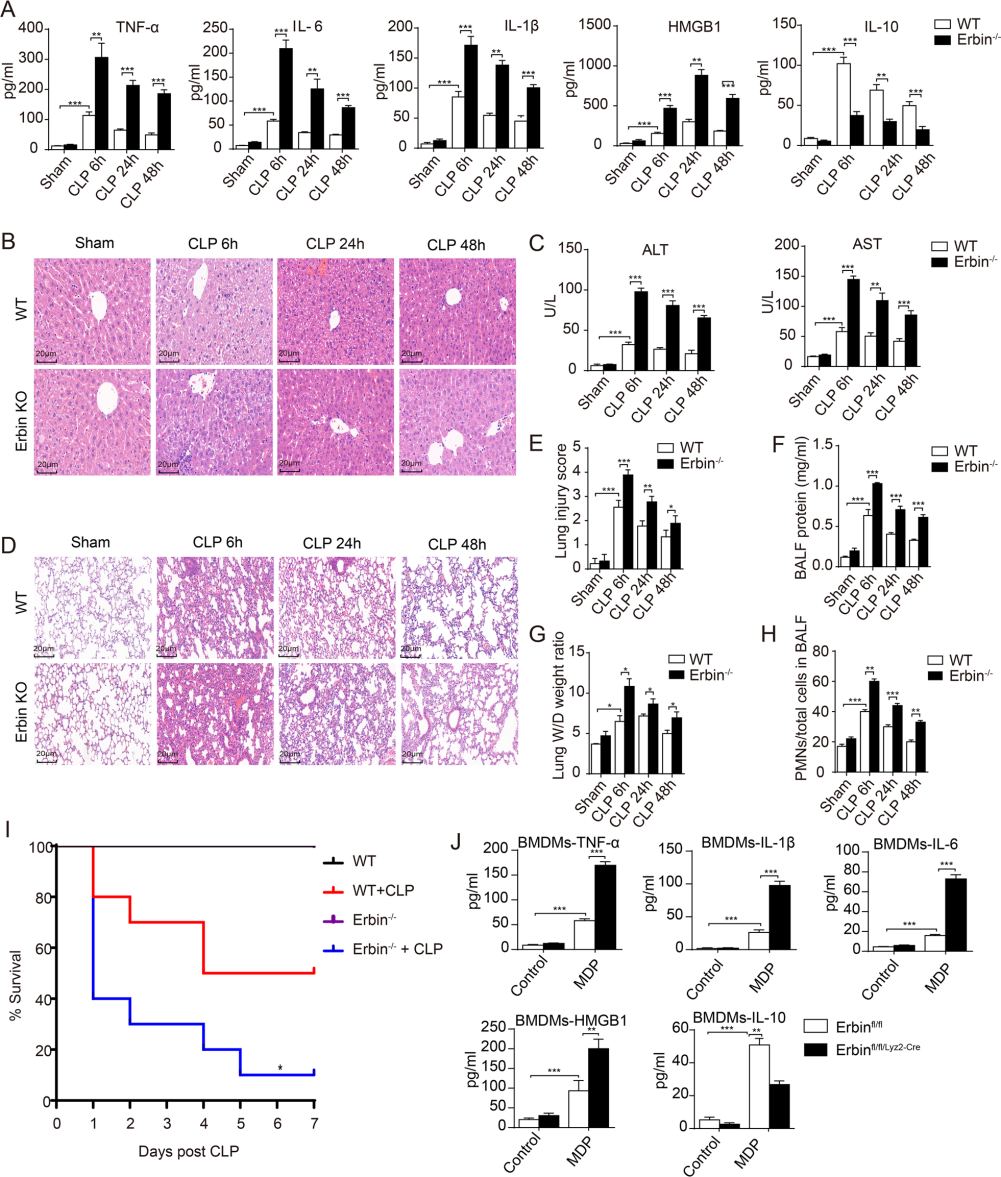
*Erbin* deficiency aggravated inflammatory response and organ injuries and deteriorated prognosis of sepsis. **A** *Erbin*^*−/−*^ mice and WT mice were subjected to CLP procedure and euthanasia of mice after CLP 6 h, 24 h, and 48h. The serum TNF-α, IL-6, IL-1β, HMGB1, and IL-10 levels were measured. **B**, **C** Histopathologic changes in the liver tissue, serum AST, and ALT levels were detected after CLP. **D**–**H** Histopathologic changes and the injury score of lung tissue, the wet/dry ratio, the protein concentration, and the PMN/total cells in BALF were measured. **I** The survival rates of WT and *Erbin*^*−/−*^ mice subjected to CLP were observed. **J** BMDMs were treated with MDP (6 h), and the TNF-α, IL-6, IL-1β, HMGB1, and IL-10 levels were measured in the supernatant of Erbin^fl/fl^ and Erbin^fl/fl/Lyz2−cre^ BMDMs. Each group had 10 mice. The data are representative of 3 independent experiments. *P < 0.05, **P < 0.01, ***P < 0.001 from ANOVA followed by Tukey’s post hoc test

To investigate the physiological role of Erbin in the inflammatory response to sepsis and sepsis-induced organ injury, we employed the CLP to establish a polymicrobial sepsis animal model, which has been widely used in previous studies [19]. As shown in Fig. 1A, CLP induced the production of many pro-inflammatory cytokines, including tumor necrosis factor-α (TNF-α), IL-6, IL-1β, and high mobility group box 1 (HMGB1) in the serum of wildtype (WT) mice, which were markedly elevated in *Erbin* knockout mice. In the same experiments, CLP-induced anti-inflammatory cytokine IL-10 production was much lower in the *Erbin* knockout CLP mice then in the WT mice. These results suggested that *Erbin* deficiency aggravated sepsis-induced inflammatory response. Furthermore, H&E staining showed that *Erbin*-knockout aggravated liver injury, which was indicated by multiple foci of hepatocyte necrosis, inflammatory cell infiltration, and fatty change in the liver of CLP mice (Fig. 1B). Consistently, the secretion of aspartate aminotransferase (AST) and alanine transaminase (ALT), which are biochemical enzymes of hepatocytes, is much increased in the serum of *Erbin* knockout mice (Fig. 1C). We also investigated the effect of *Erbin*-knockout on lung histopathology in sepsis mice. H&E staining showed that CLP-induced severe pathological changes, including pulmonary capillary congestion, pulmonary interstitial edema, mass inflammatory cells infiltration into the alveolar space and lung interstitium, as well as alveolar wall thickening in WT mice, which were dramatically enhanced in the lung tissues of CLP-challenged *Erbin* knockout mice (Fig. 1D). Moreover, we have also employed a scoring system to assess the degree of lung injury [20]. As shown in Fig. 1E, the quantitative scoring of histological lung injury in *Erbin* knockout mice was markedly increased compared with that in WT mice after CLP. Additionally, the BALF protein concentration and wet/dry ratio, two widely used indicators of pulmonary vascular permeability, were significantly increased in CLP-challenged *Erbin* knockout mice compared to those sham WT mice (Fig. 1F, G). We also detected the ratio of the number of polymorphonuclears (PMNs) relative to the total cells in the BALF. Compared to the WT mice, the PMNs/total cells ratio in *Erbin* knockout mice was dramatically increased after CLP (Fig. 1H). These results suggested that *Erbin*-deficiency triggers aggravated liver and lung injuries in CLP-challenged mice. Finally, we examined the rates of CLP-induced septic lethality of both WT and *Erbin* knockout mice, showing that *Erbin* deficiency decreased the survival rate of sepsis mice (Fig. 1I).

In vitro, we isolated BMDMs from Erbin^fl/fl^ and Erbin^fl/fl/Lyz2−Cre^ mice and primed the cells with MDP for 6 h. Similar to in vivo results, the pro-inflammatory cytokines TNF-α, IL-6, IL-1β, HMGB1, and the anti-inflammatory cytokine IL-10 levels were increased in the MDP-treated wild-type supernatant BMDMs, and *Erbin* deficiency increased the pro-inflammatory cytokines and decreased the IL-10 production (Fig. 1J). All these results suggested that *Erbin* deficiency increased sepsis-induced inflammatory response, aggravated organ injuries, and reduced survival rates in CLP-challenged mice, indicating an anti-inflammatory protective role of Erbin in sepsis and MDP-treated BMDMs. 

### *Erbin* deficiency exacerbates autophagy impairment in sepsis mice and MDP-treated BMDMs

**Fig. 2:**
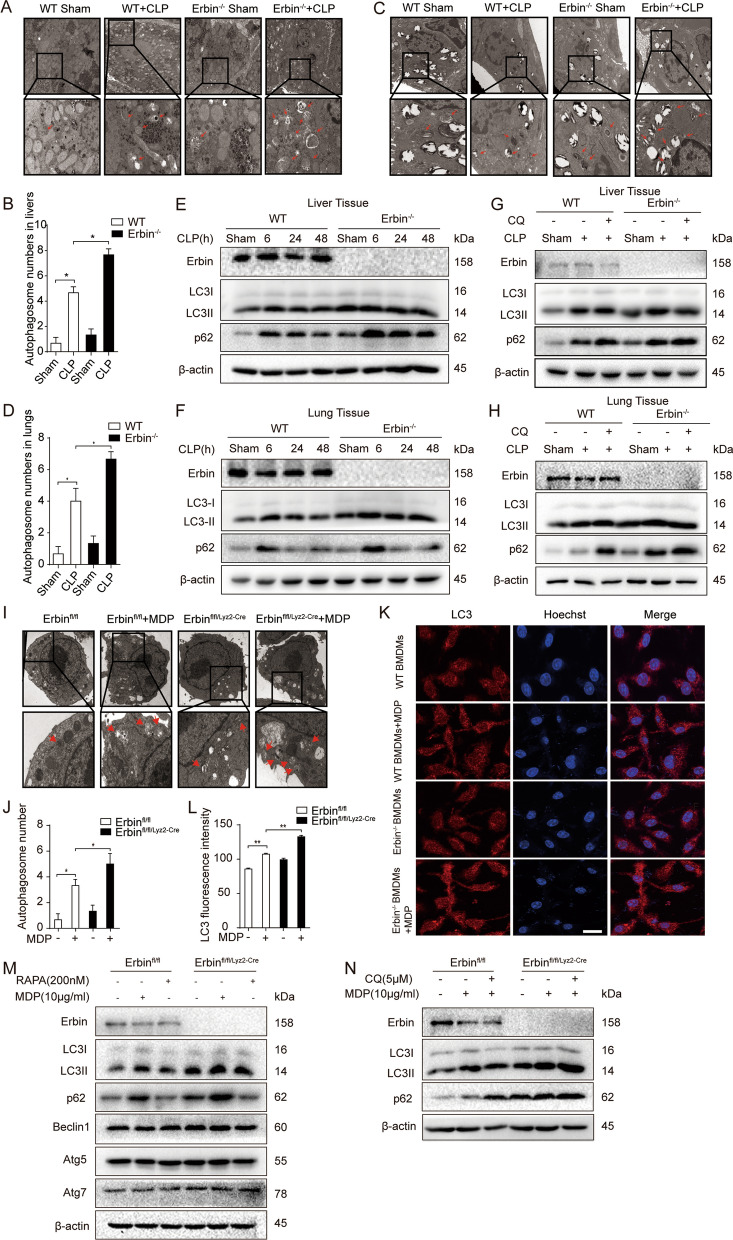
*Erbin* deficiency exacerbates autophagy impairment in sepsis mice and MDP-treated BMDMs. **A, C** WT and *Erbin*^*−/−*^ mice were subjected to CLP 6 h, and the mouse liver and lung tissues were examined by transmission electron microscopy (TEM). **B, D** Autophagosomes were quantified by analysis of (**A**) and (**C**) results. The arrow indicates autophagosomes. Scale bar: 1 μm. **E** Western blotting was used to estimate the protein levels of LC3-I/II and p62 at 6 h, 24 h, and 48 h after CLP in mice liver tissue. **F** Western blotting was used to estimate the protein levels of LC3-I/II and p62 at 6 h, 24 h, and 48 h after CLP in mice lung tissue. **G** CQ (60 mg/kg) was used as a pretreatment before CLP to inhibit autophagosome-lysosome fusion. The protein expression of LC3-I/II and p62 in liver tissues were detected by western blotting. **H** CQ (60 mg/kg) was used as a pretreatment before CLP to inhibit autophagosome-lysosome fusion. The protein expression of LC3-I/II and p62 in lung tissues was detected by western blotting. **I** BMDMs were examined by transmission electron microscopy (TEM). The arrow indicates autophagosome. Scale bar: 1 μm. **J** Autophagosomes were quantified by analysis of results of (**I**). **K** The LC3-I/II fluorescence was detected by confocal microscopy, scale bar: 50μm. **L** The LC3-I/II fluorescence staining was quantified by analysis of results of (**K**). **M** Erbin^fl/fl^ and Erbin^fl/fl/Lyz2−cre^ BMDMs were treated with MDP (6 h), and the western blotting was used to estimate the protein levels of LC3-I/II, p62, Beclin1, ATG5, and ATG7. 200 nM RAPA was used as a positive control for autophagy. **N** CQ (5 μM) was used as a pretreatment before MDP to inhibit autophagosome-lysosome fusion. The LC3-I/II conversion and p62 accumulation were detected by western blotting in BMDMs. *P < 0.05, **P < 0.01, ***P < 0.001 from ANOVA followed by Tukey’s post hoc test

To reveal the further mechanism of Erbin in sepsis, we investigate the effects of Erbin on autophagy in polymicrobial sepsis mice and MDP-treated BMDMs. The liver and lung tissues were dissected at 6 h, 24 h, and 48 h after CLP. Transmission electron microscopy (TEM) experiments showed that more autophagosomes were observed in the liver or lung of *Erbin* knockout CLP mice than WT CLP (Fig. 2A–D). Furthermore, we have examined the protein levels of several critical components in the autophagy pathway. The results showed that CLP increased LC3-II and SQSTM1 (p62) protein levels in both liver and lung of WT mice, which further accumulated in *Erbin*-knockout mice (Fig. 2E, F). Generally, the increased conversion of LC3-I to LC3-II represents the enhancement of autophagy. However, the failure of degradation of LC3-II at the lysosomal stage could also lead to the accumulation of LC3-II, resulting in the illusion that autophagy flow is blocked or autophagy is enhanced. De Wet et al. revealed that the increase of p62 level could be detected in autophagy-deficient tissue cells, indicating that the decrease of p62 level accompanies the enhancement of autophagy, but the inhibition of autophagy increases p62 instead [21]. Therefore, we evaluated autophagy status by treating with rapamycin (RAPA, an autophagy agonist) or CQ, a lysosomal inhibitor, and detected LC3-II and p62 protein levels, respectively. CQ treatment increased the level of LC3-II and p62 in liver or lung tissues compared with CLP mice. Compared with WT CLP mice treated with CQ, *Erbin* knockout caused a further increased LC3-II or p62 in the liver and lung tissues of CLP mice (Fig. 2G, H).

Additionally, in vitro experiments, Erbin^fl/fl^ and Erbin^fl/fl/Lyz2−Cre^ BMDMs were exposed to 10 μg/ml MDP for 6 h. The TEM experiments showed that MDP treatment increased autophagosome numbers in Erbin^fl/fl^ BMDMs, and *Erbin*-knockout caused further more autophagosome numbers in MDP-treated BMDMs (Fig. 2I, J). Immunofluorescence experiments also showed MDP treatment increased LC3-I/II staining in Erbin^fl/fl^ BMDMs, and the LC3-I/II staining was further increased after *Erbin* ablation in MDP-induced BMDMs (Fig. 2K). We used RAPA as positive control, results showed that RAPA could induce complete autophagic flux in BMDMs, with the increased levels of LC3II and the decreased levels of p62. However, MDP treatment increased the protein levels of LC3-II as well as p62, and *Erbin*-knockout caused further accumulation of LC3-II and p62 proteins after MDP treatment, with no significant changes in AGT5, ATG7, Beclin1 protein levels (Fig. 2M). To study autophagic flux, CQ was used to inhibit autophagy, results showed that the levels of LC3-II and p62 markedly increased in MDP + CQ treated Erbin^fl/fl/Lyz2−Cre^ BMDMs than those in MDP + CQ treated Erbin^fl/fl^ BMDMs (Fig. 2N). Those results indicated that Erbin deficiency could result in lysosomal dysfunction, damaged autophagosomes degradation and the accumulation of LC3-II and p62 proteins.

### Erbin alleviates sepsis-induced inflammatory response and organ injury by regulating the autophagy-lysosome pathway (ALP)

**Fig. 3:**
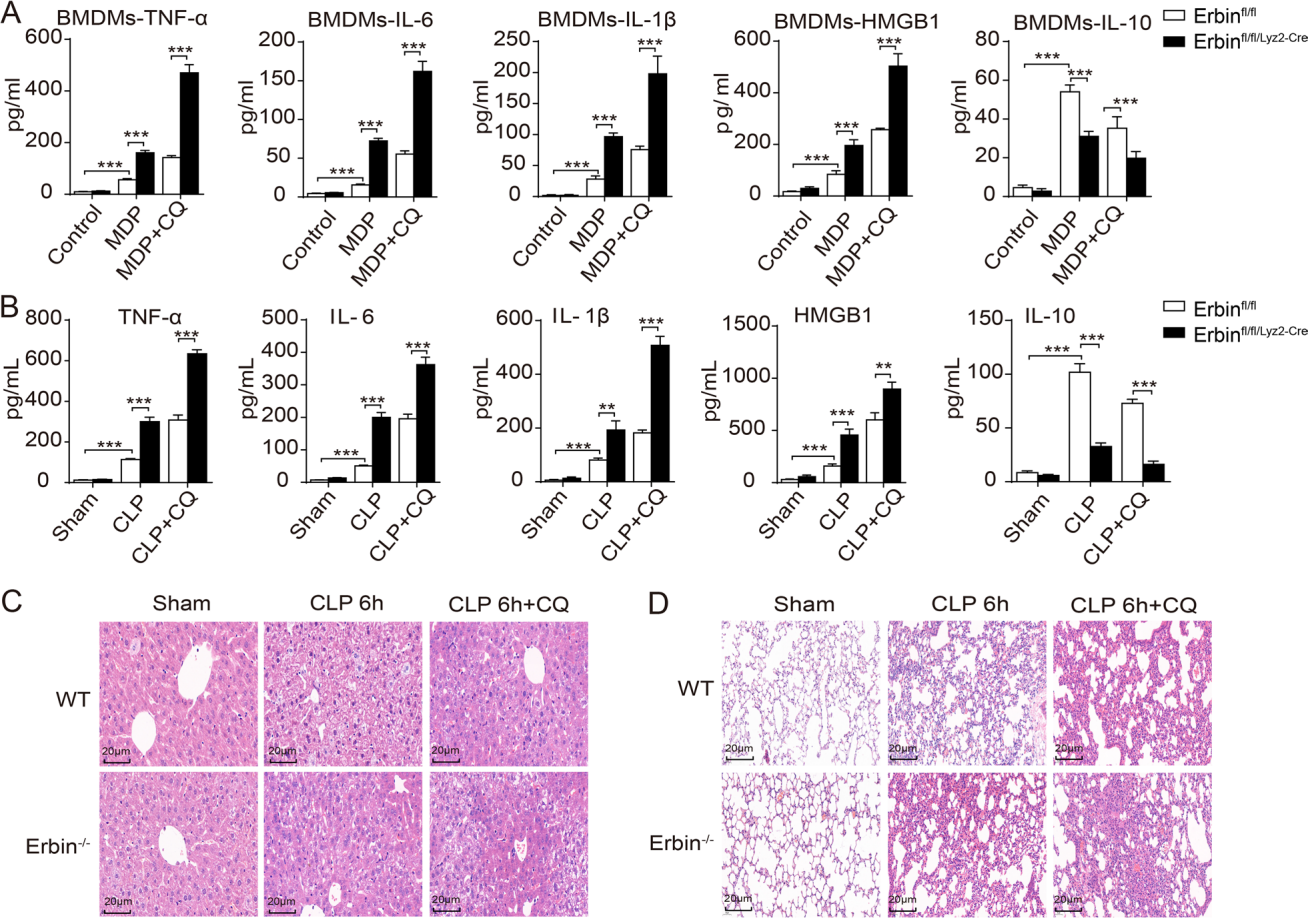
Erbin-mediated autophagy response alleviated sepsis-induced inflammatory response and organ injury. **A** Erbin^fl/fl^ and Erbin^fl/fl/Lyz2−cre^ BMDMs were pretreated with CQ (5 μM) for 12 h and then treated with MDP for 6 h. The TNF-α, IL-6, IL-1β, HMGB1, and IL-10 levels were measured in the supernatant of Erbin^fl/fl^ and Erbin^fl/fl/Lyz2−cre^ BMDMs. **B** The Erbin^−/−^ and WT littermate mice were pretreated with CQ (60 mg/kg) before CLP 1 h, and mice were euthanized after CLP 6 h. The serum TNF-α, IL-6, IL-1β, HMGB1 and IL-10 levels were measured by ELISA. **C**, **D** Histopathologic changes in the liver and lung tissues were detected by H&E staining. Each group had 10 mice. Data shown are representative of 3 independent experiments. *P < 0.05, **P < 0.01, ***P < 0.001 from ANOVA followed by Tukey’s post hoc test

To determine whether Erbin-mediated autophagy regulates sepsis-induced inflammatory response and organ injury, we investigated the level of inflammatory cytokines TNF-α, IL-6, IL-1β, HMGB1, and IL-10 in polymicrobial sepsis mice and MDP-treated BMDMs with CQ treatment. The CQ treatment caused a further aggravated inflammatory response, as indicated by the increased levels of pro-inflammatory cytokines of TNF-α, IL-6, IL-1β, and HMGB1, and the decreased levels of anti-inflammatory cytokine IL-10. Importantly, Erbin deficiency further increased the inflammatory response via CQ treatment in vitro and vivo compared with Erbin^fl/fl^ BMDMs or WT CLP mice treated with CQ, respectively (Fig. 3A, B). The H&E staining experiments showed that the inhibition of autophagy by CQ treatment caused severe injury of the liver and lung tissues of WT mice after the CLP challenge, which the injury scores were dramatically aggravated in the liver and lung tissues of CLP-challenged *Erbin* knockout mice (Fig. 3C, D). Those observations indicated that Erbin promoted the autophagy process and alleviated sepsis-induced inflammatory response and organ injury.

### Erbin improves lysosomal biogenesis in MDP-treated BMDMs

**Fig. 4:**
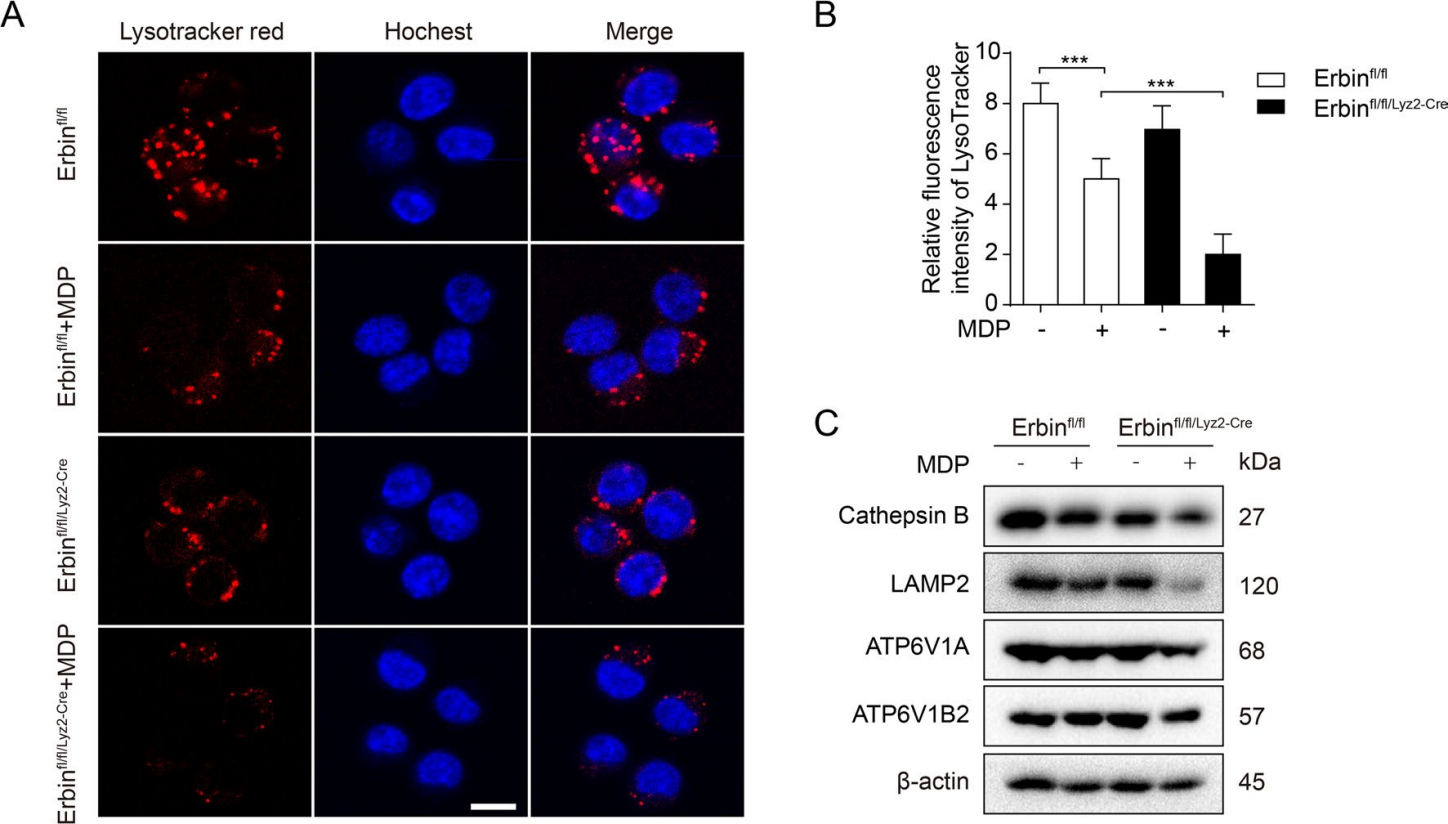
Erbin enhanced lysosome biogenesis in BMDMs. **A** BMDMs were treated with 10 μg/ml MDP (6 h) and then stained with LysoTracker Red for 15 min. The fluorescence intensity of treated cells was measured by confocal microscopy. Scale bar: 25 μm. **B** Fluorescence puncta were quantified by analysis of results of (**A**). **C** Erbin^fl/fl^ and Erbin^fl/fl/Lyz2−cre^ BMDMs were treated with MDP for 6 h, and western blotting was used to estimate the protein levels of LAMP2, CTSB, ATP6V1A, and ATP6V1B2. *P < 0.05, **P < 0.01, ***P < 0.001 from ANOVA followed by Tukey’s post hoc test

To further explore the regulatory mechanism of Erbin in autophagy process during sepsis. We detected the lysosomal acidic microenvironment by using LysoTracker Red stain. Compared with MDP-treated Erbin^fl/fl^ BMDMs, the fluorescence intensity was decreased in MDP-treated *Erbin*-knockout BMDMs, indicating that *Erbin* deficiency reduced the acidification of lysosomes (Fig. 4A, B). Interestingly, we detected the protein levels of vacuolar-type proton pumping ATPase (V-ATPase) subunits ATP6V1A and ATP6V1B2. Compared with MDP-treated Erbin^fl/fl^ BMDMs, the ATP6V1A and ATP6V1B2 levels were reduced in MDP-treated Erbin^fl/fl/Lyz2−cre^ BMDMs. The levels of lysosome proteins lysosome-associated membrane protein-2 (LAMP-2) and CTSB were significantly decreased in MDP-treated Erbin^fl/fl/Lyz2−cre^ BMDMs than that in MDP-treated Erbin^fl/fl^ BMDMs (Fig. 4C). Those results indicated that *Erbin* deficiency decreased lysosome numbers and lysosomal degradative function.

### Erbin promotes TFEB nuclear translocation and transcription activity

**Fig. 5:**
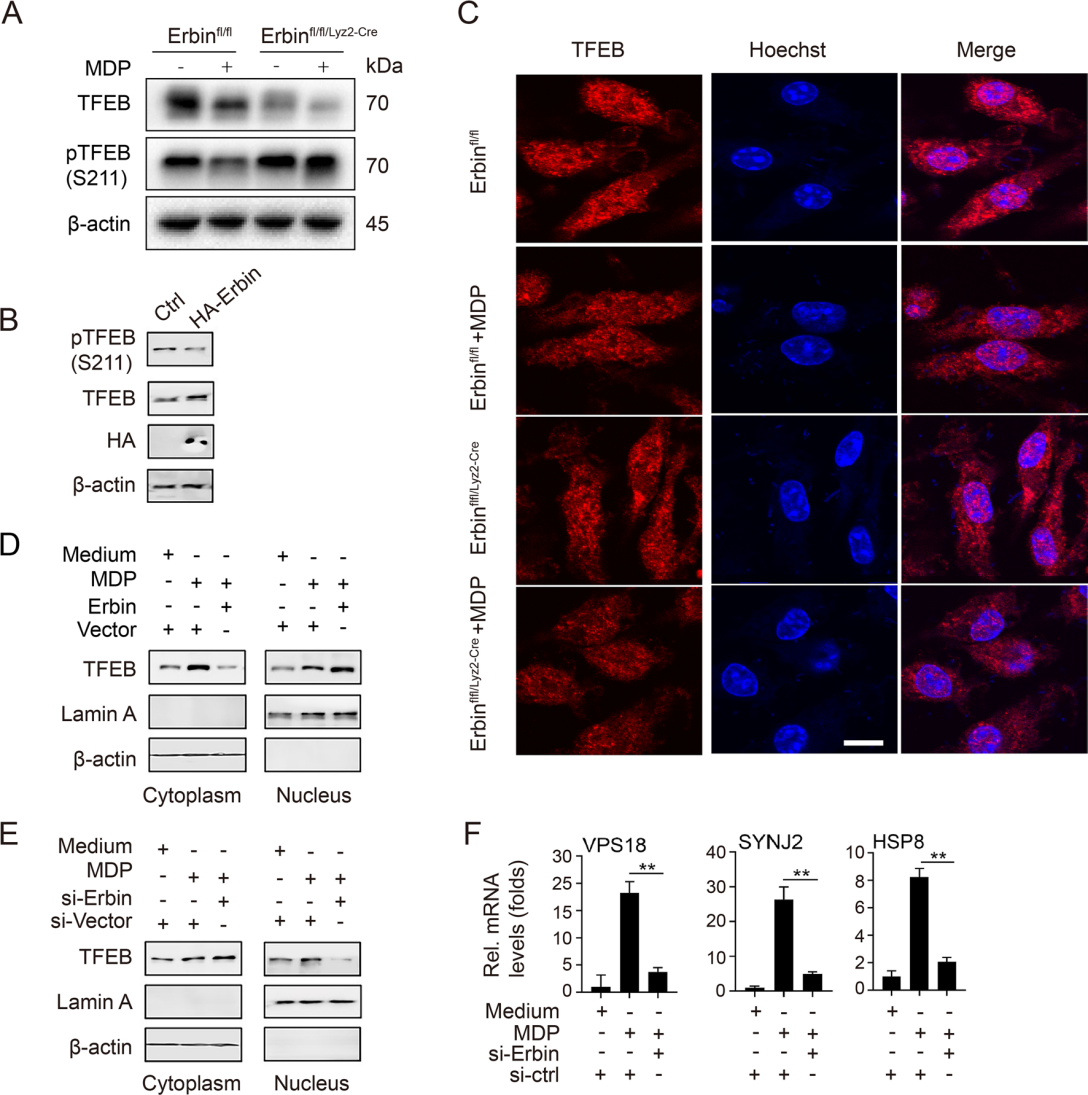
Erbin promotes TFEB nuclear translocation and transcription activity. **A** Erbin^fl/fl^ and Erbin^fl/fl/Lyz2−cre^ BMDMs were treated with MDP for 6 h, and western blotting was used to estimate the protein levels of total TFEB and pTFEB (S211). **B** THP-1 macrophages were transfected with vector control or HA-Erbin for 36 h prior to western blot assays. **C** BMDMs were treated with 10μg/ml MDP for 6 h and then incubated with TFEB antibody. The fluorescence intensity of treated cells was measured by confocal microscopy. Scale bar: 25 μm. **D** THP-1 macrophages were transfected with vector control or HA-Erbin for 36 h. Then, cells were treated with or without MDP (10 μg/ml) for 6 h. Cytosolic and nuclear extracts were prepared and subjected to western blot analyses. Lamin A and β-actin were used as the internal control for nuclear and cytosolic fractions, respectively. **E** Experiments were performed similarly to those in **D**, except indicated siRNA was used. **F** THP-1 macrophages were transfected with siRNA control or si-Erbin for 36 h and treated with or without MDP (10 μg/ml) for 6 h before qPCR analyses. The data shown are representative of 3 independent experiments

TFEB is located in the cytoplasm in the form of phosphorylation, and dephosphorylated TFEB transfers from cytoplasmic into intracellular nucleus, activating the expression of lysosomal and autophagy-related genes. We found that *Erbin* knockout increased the phosphorylated TFEB/S211 level in MDP-treated Erbin^fl/fl/Lyz2−cre^ BMDMs compared with MDP-treated Erbin^fl/fl^ BMDMs group, and *Erbin* over-expression inhabited the phosphorylated TFEB/S211 level in THP-1macrophages (Fig. 5A, B). Consistently, the subcellular fractionation experiments showed that *Erbin* over-expression increased the cytoplasm-to-nucleus translocation of TFEB, and *Erbin* knockdown decreased the nuclear translocation of TFEB after MDP stimulation (Fig. 5C–E). These results indicated that Erbin promotes the TFEB nuclear translocation. The mRNA levels of the TFEB target genes VPS18, SYNJ2, and HSP8 were significantly decreased in MDP-treated *Erbin*-knockdown THP-1 macrophages compared to the control group (Fig. 5F). Those results suggested that Erbin promotes TFEB nuclear translocation and transcription activity.

### Erbin binds to TFEB and regulates the stability of TFEB-14-3-3 and TFEB-PPP3CB complexes

**Fig. 6:**
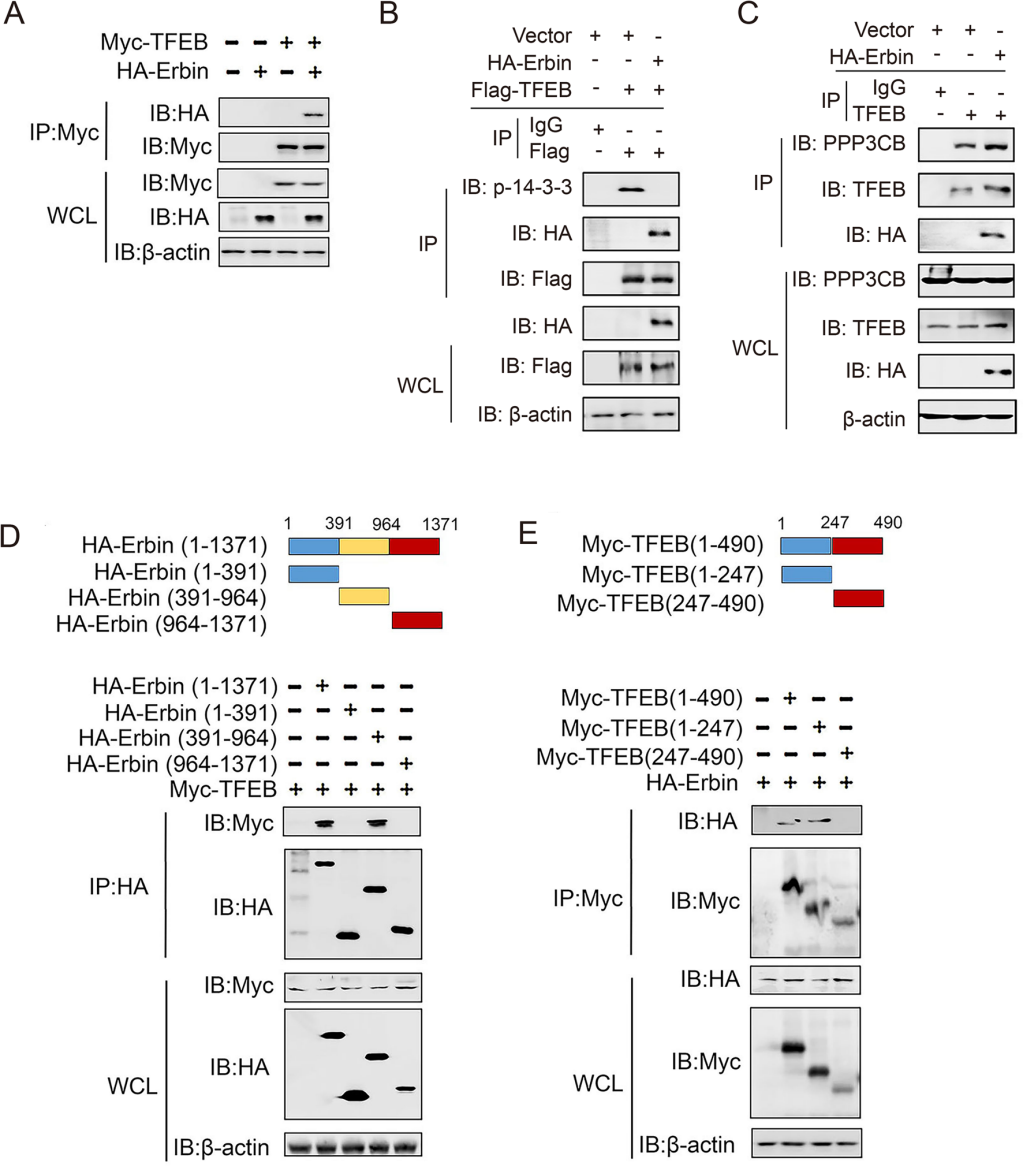
Erbin binds to TFEB and regulates the stability of TFEB-14-3-3 and TFEB-PPP3CB complexes. **A** 293T cells were transfected with indicated plasmids. 48 h post-transfection, Co-IP and immunoblot analysis were performed with indicated antibodies. **B**, **C** Experiments were performed similarly to those in **A**, except indicated plasmids were used. **D** Schematic diagram of the full-length and truncated constructs of Erbin. The 293T cells were co-transfected with Myc-TFEB and the indicated truncated Erbin constructs for 48 h. Co-immunoprecipitation and immunoblot analyses were performed with the indicated antibodies. **E** Schematic diagram of the full-length and truncated constructs of TFEB. The 293T cells were co-transfected with HA-Erbin and the indicated truncated TFEB constructs for 48 h. Co-immunoprecipitation and immunoblot analyses were performed with the indicated antibodies

Except for ATG proteins involved in the regulation of autophagy, several diverse factors are involved in the regulation of autophagy-lysosome pathway, including TFEB and lysosome-associated proteins in sepsis [16]. To explore how Erbin regulates lysosomal biogenesis, we investigated whether Erbin associates with TFEB. We transferred several plasmids in 293T cells, we found that Erbin strongly interacted with TFEB (Fig. 6A). To explore the mechanism of Erbin-regulated TFEB cytoplasm-to-nucleus translocation, we transfected with HA-Erbin and Flag-TFEB in 293T cells, immunoprecipitation indicated that TFEB could combine with 14-3-3, and Erbin co-expression impaired TFEB-14/3/3 complex formation (Fig. 6B). Meanwhile, we transferred HA-Erbin in 293T cells, and we found that TFEB could combine with PPP3CB, and Erbin co-expression enhanced the interaction between TFEB and PPP3CB (Fig. 6C). The 293T cells were co-transfected with Myc-TFEB and the indicated truncated Erbin constructs, the further domain mapping experiments showed that the C-terminal of Erbin (aa 391–964) was required for binding Erbin to TFEB. Additionally, TFEB interacted with Erbin via its N-terminal (aa 1–247) (Fig. 6D, E). Thus, these results suggest that Erbin regulates autophagy through TFEB-14-3-3 and TFEB-PPP3CB complexes.

## Discussion

**Fig. 7:**
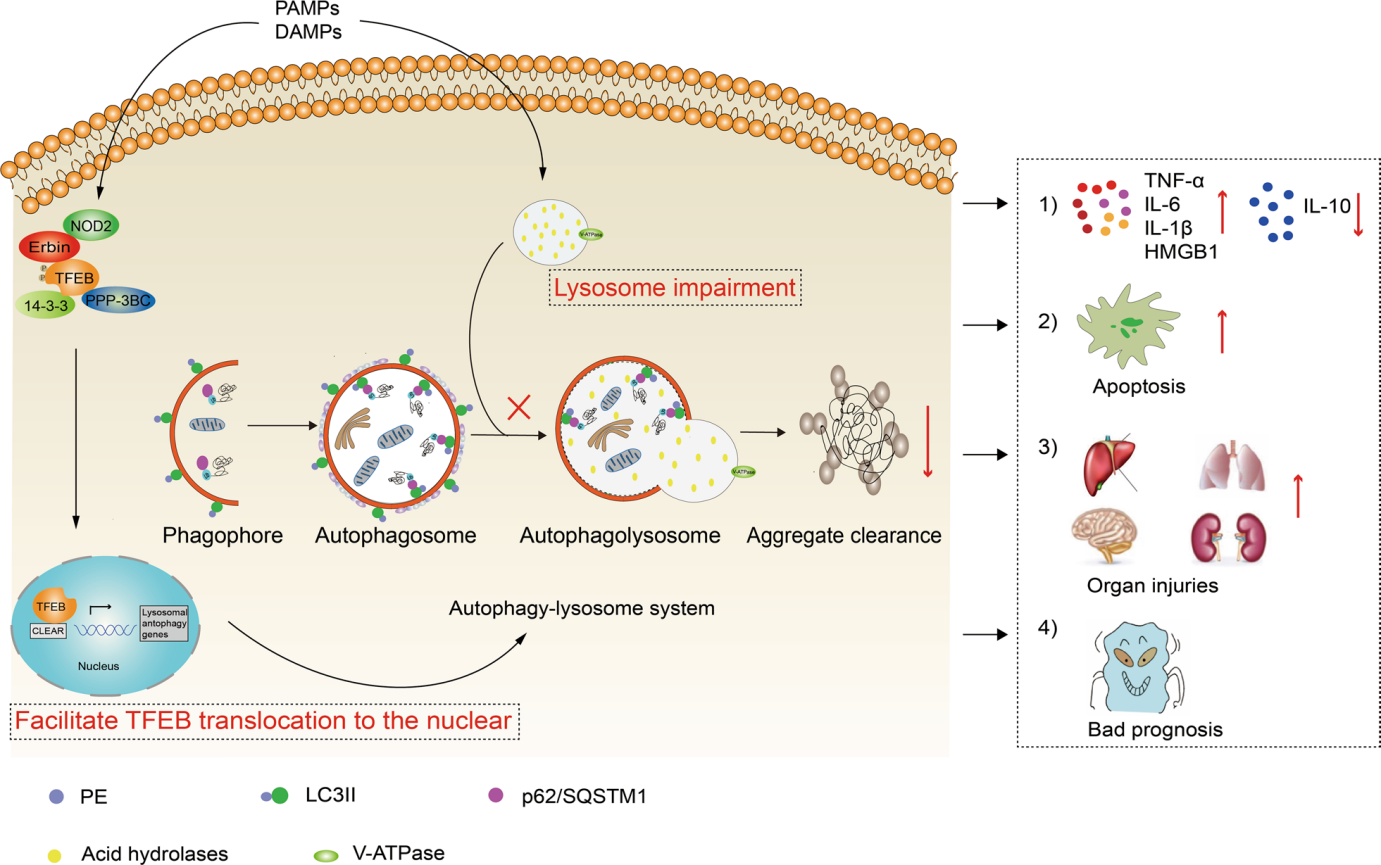
Erbin rescues sepsis-induced ALP dysfunction by binding to TFEB, alleviating inflammation response and organ injuries. In this model, the NOD2-NF-κB pathway is activated on DAMPs and PARMs stimulation, resulting in the dysfunction of ALP, especially the disrupted lysosomal acidic environment. In this process, Erbin physically interacts with TFEB and regulates TFEB-14-3-3 and TFEB-PPP3BC complexes stability, which regulates the TFEB nuclear translocation, thus regulating TFEB transcription levels and improving lysosomal biogenesis. Thus, impaired Erbin-mediated ALP leads to an aggravated sepsis inflammation response and a deteriorated prognosis. Those suggested that Erbin's targeting of TFEB might rescue the dysfunctional ALP and alleviate the inflammation response and organ injuries following sepsis

The present study demonstrates that *Erbin* deficiency disrupts autophagy progress, thus amplifying the inflammatory response and organ injury during sepsis. Importantly, its impaired acidic microenvironment of lysosomes accounted for the autophagy dysfunction. Mechanically, Erbin promotes lysosomal biogenesis and autophagy through directly targeting TFEB and regulating TFEB-14-3-3 and TFEB-PPP3CB complexes, thus alleviating inflammatory responses and organ injuries. Results obtained in the current study are represented schematically in Fig. 7.

Erbin binds to NOD2, one of the PRRs, and detects PGN through recognizing of MDP common in the gram-positive and gram-negative bacterium and then up-regulation of pro-inflammatory molecules. McDonald et al. found that Erbin deletion led to increased sensitivity of mouse fibroblasts (MEFs) to MDP, as shown by a significant increase in cytokine MCP-1 levels after Erbin deletion [17]. Shen et al. found that Erbin-deficient mice exhibited a more severe inflammatory response and intestinal barrier disruption of colitis mice [22]. Here, we found that *Erbin* deficiency aggravated sepsis-induced inflammatory responses, organ dysfunction, and even death. Therefore, Erbin might be an early warning indicator for sepsis, which uncovers the therapeutic potential of Erbin in sepsis.

Autophagy serves as an immune response to microbial infection [23], and several previous investigations have suggested autophagy activity is increased in response to sepsis insult based on the increased autophagosomes and the conversion of LC3-I to LC3-II. However, this assumption is not necessarily correct, as the presence of numerous autophagosomes or the increased LC3-II could result from an increase in formation or a decrease in clearance [24]. For example, in a pancreatitis model, the autophagosome formation and LC3-I/II were significantly increased after LPS treatment. However, this resulted from the blockade fusion of autophagosomes and lysosomes rather than the induction of autophagy [25]. SQSTM1/p62, a stress-induced cellular protein, acts as a receptor for selective autophagy and is considered to be a good reflection of clearance function, with the LIR domain interacting with multiple sites of LC3, and subsequently degraded along with LC3 and the ubiquitinated protein aggregates [26]. Thus, failing to efficiently degrade p62 suggested that autophagy should not be sufficient, possibly due to impaired lysosomal/autolysosomal functions or decreased lysosome numbers. In the present study, we found that Erbin alleviated the dysfunction of ALP caused by sepsis, which may account for the protective role of Erbin in sepsis.

Autophagy dysfunction is considered the potential toxic mechanism of sepsis. Saitoh et al. reported that *Atg16L1* deficiency increased caspase-1 activation and IL-1β processing in LPS-stimulated macrophages [27]. Similarly, after endotoxin stimulation, IL-1β production in ATG7-deficient or treated with autophagy inhibitor 3-methyladenine (3-MA) macrophages is enhanced. Chung et al. reported that pretreatment of chloroquine aggravated LPS-induced lipid accumulation and inflammation in C57BL6 mouse livers [28]. Autophagic inducer RAPA improved the survival rate, histologic scores, lung wet/dry weight ratio, PaO_2_/FiO_2_, MPO activity, the pro-inflammatory cytokines TNF-α, HMGB1, IL-6, IL-10, and MCP1 production in septic mice, but there were exacerbated above indicators in sepsis mice pretreated with autophagy inhibitor 3-MA [29]. Functionally, in our study, expression patterns of inflammatory cytokine and organ injuries were significantly increased in *Erbin* deficiency mice after being treated with CQ. Higher expression of TNF-α, IL-6, IL-1β, and HMGB1 accompanied with aggravated organ injuries were observed in *Erbin* deficiency after CQ treatment. It indicated that Erbin alleviated sepsis-induced inflammatory responses and organ injuries by rescuing autophagic flux impairment.

Since autophagy is a lysosome-dependent degradation process that includes the early stage of autophagy initiation and the late stage of autophagy degradation [30]. Fusion of autophagosomes with lysosomes is necessary for complete autophagic flux [31]. Lysosome plays a central role in ALP. The destruction of lysosomes comprises two sides named lysosomal membrane permeabilization (LMP) and lysosomal membrane rupture (LMR) [32]. Mutation of LAMP-1 and LAMP-2 influenced autophagosomes’ combination with endosomal vesicles to lysosomes, and lysosomal proteases cathepsins are required for subsequent substrate degradation [33]. Each of these lysosomal behaviors is influenced by the intraluminal pH of the lysosome, which is maintained in the low acidic range by a proton pump, the vacuolar ATPase (v-ATPase) [34]. In Erbin deficiency BMDMs, we found lower levels of CTSB and LAMP2, as well as a more serious disrupted lysosome acidic environment, than the MDP-treated WT BMDMs. These results suggested that Erbin deficiency would destroy the lysosome structure and lysosomal biogenesis to influence the autophagic flux.

Additionally, there are other genes that affect multiple steps of this progress. TFEB was recently discovered as a major regulator of the ALP as well as a potential therapeutic target [35]. In addition, PPP3CB-mediated dephosphorylation of TFEB, and YWHA/14-3-3 dissociation were required for TFEB nuclear translocation [36]. In the present study, total TFEB expression was increased in response to MDP treatment. Additionally, *Erbin* deficiency resulted in a marked accumulation of TFEB protein in the cytoplasmic subfraction with a corresponding decline in the nuclear subfraction. We also found that *Erbin* overexpression promoted TFEB translocation from the cytoplasm to the nucleus after MDP treatment in vitro. Furthermore, co-immunoprecipitation showed that Erbin promoted the dissociation of TFEB from the TFEB-14-3-3 complex and enhanced the interaction between TFEB and PPP3CB. The finding suggested that Erbin-mediated TFEB translocation to the nuclear through the regulation of TFEB-14-3-3 and TFEB-PPP3CB complexes stability. It uncovered the potential molecular mechanism of Erbin regulating autophagy after sepsis insult.

In conclusion, our study demonstrated that Erbin promoted the autophagy process and activated lysosomal biogenesis via direct targeting and activation of TFEB. The cooperatively molecular action rescued autophagy dysfunction caused by sepsis insult and alleviated inflammatory response and organ injuries during the sepsis challenge. Our findings thus reveal novel insight into the regulatory mechanisms of Erbin on autophagy and inflammatory response and provide a potential therapeutic target.

## Data Availability

The datasets generated during and/or analyzed during the current study are available from the corresponding author on reasonable request.

## Abbreviations

TFEB: Transcription factor EB
ALP: Autophagy-lysosome pathway
BMDMs: Bone marrow-derived macrophages
MDP: Muramyl dipeptide
LPS: Lipopolysaccharide
CLP: Cecal ligation and puncture
DSS: Dextran sulfate sodium
NOD2: Nucleotide-binding oligomerization domain-containing protein 2
NLRP3: NOD-like receptor thermal protein domain associated protein 3
LRRs: Leucine-rich repeats
DAMP: Danger-associated molecular pattern
PAMP: Pathogen-associated molecular patterns
PGN: Peptidoglycan
CQ: Chloroquine
RAPA: Rapamycin
BALF: Bronchoalveolar lavage fluid
PMN: Polymorphonuclear
PFA: Paraformaldehyde
BSA: Bovine serum albumin
PVDF: Polyvinylidene difluoride
ELISA: Enzyme-linked immunosorbent assay
H&E: Hematoxylin and eosin
WT: Wildtype
AST: Aspartate aminotransferase
ALT: Alanine transaminase
IL: Interleukin
TNF-α: Tumor necrosis factor-α
HMGB1: High mobility group box 1
3-MA: 3-Methyladenine
qPCR: Quantitative polymerase chain reaction
TEM: Transmission electron microscopy
MEF: Mouse fibroblast
TLR: Toll-like receptor
LC3: Microtubule-associated protein 1 light chain 3
ATG: Autophagy-related gene
CTSB: Cathepsin B
LMP: Lysosomal membrane permeabilization
LMR: Lysosomal membrane rupture
v-ATPase: Vacuolar ATPase

## References

[1] Levine B, Kroemer G (2019). Biological functions of autophagy genes: a disease perspective. Cell.

[2] Jang YJ, Kim JH, Byun S (2019). Modulation of autophagy for controlling immunity. Cells.

[3] Deretic V (2021). Autophagy in inflammation, infection, and immunometabolism. Immunity.

[4] Dikic I, Elazar Z (2018). Mechanism and medical implications of mammalian autophagy. Nat Rev Mol Cell Biol.

[5] Settembre C, Fraldi A, Medina DL, Ballabio A (2013). Signals from the lysosome: a control centre for cellular clearance and energy metabolism. Nat Rev Mol Cell Biol.

[6] Ballabio A, Bonifacino JS (2020). Lysosomes as dynamic regulators of cell and organismal homeostasis. Nat Rev Mol Cell Biol.

[7] Mahapatra KK, Mishra SR, Behera BP, Patil S, Gewirtz DA, Bhutia SK (2021). The lysosome as an imperative regulator of autophagy and cell death. Cell Mol Life Sci.

[8] Xia Q, Wu X, Rong K, Zhou Z, Li X, Fei T (2020). Lysosomal autophagy promotes recovery in rats with acute knee injury through TFEB mediation. J Orthop Surg Res.

[9] Yim WW, Mizushima N (2020). Lysosome biology in autophagy. Cell Discov..

[10] Song XB, Liu G, Liu F, Yan ZG, Wang ZY, Liu ZP (2017). Autophagy blockade and lysosomal membrane permeabilization contribute to lead-induced nephrotoxicity in primary rat proximal tubular cells. Cell Death Dis.

[11] Xia Y, Liu N, Xie X, Bi G, Ba H, Li L (2019). The macrophage-specific V-ATPase subunit ATP6V0D2 restricts inflammasome activation and bacterial infection by facilitating autophagosome-lysosome fusion. Autophagy.

[12] Puertollano R, Ferguson SM, Brugarolas J, Ballabio A (2018). The complex relationship between TFEB transcription factor phosphorylation and subcellular localization. EMBO J.

[13] Xu Y, Ren J, He X, Chen H, Wei T, Feng W (2019). YWHA/14–3–3 proteins recognize phosphorylated TFEB by a noncanonical mode for controlling TFEB cytoplasmic localization. Autophagy.

[14] Chen M, Dai Y, Liu S, Fan Y, Ding Z, Li D (2021). TFEB biology and agonists at a glance. Cells.

[15] Kumar S, Jain A, Choi SW, Peixoto Duarteda Silva G, Allers L, Mudd MH (2020). Mammalian Atg8-family proteins are upstream regulators of the lysosomalsystem by controlling MTOR and TFEB. Autophagy.

[16] Borg JP, Marchetto S, Le Bivic A, Ollendorff V, Jaulin-Bastard F, Saito H, Fournier E, Adélaïde J, Margolis B, Birnbaum D (2022). ERBIN: a basolateral PDZ protein that interacts with the mammalian ERBB2/HER2 receptor. Nat Cell Biol.

[17] McDonald C, Chen FF, Ollendorff V, Ogura Y, Marchetto S, Lecine P (2005). A role for Erbin in the regulation of Nod2-dependent NF-kappaB signaling. J Biol Chem.

[18] Eitel J, Krull M, Hocke AC, N'Guessan PD, Zahlten J, Schmeck B (2008). Beta-PIX and Rac1 GTPase mediate trafficking and negative regulation of NOD2. J Immunol.

[19] Rittirsch D, Huber-Lang MS, Flierl MA, Ward PA (2009). Immunodesign of experimental sepsis by cecal ligation and puncture. Nat Protoc.

[20] Kangelaris KN, Calfee CS, May AK, Zhuo H, Matthay MA, Ware LB (2014). Is there still a role for the lung injury score in the era of the Berlin definition ARDS?. Ann Intensive Care.

[21] de Wet S, Du Toit A, Loos B (2021). Spermidine and rapamycin reveal distinct autophagy flux response and cargo receptor clearance profile. Cells.

[22] Shen T, Li S, Cai L-D, Liu J-L, Wang C-Y, Gan W-J, Li X-M, Wang J-R, Sun L-N, Deng M, Liu Y-H, Li J-M (2018). Erbin exerts a protective effect against inflammatory bowel disease by suppressing autophagic cell death. Oncotarget.

[23] Deretic V, Saitoh T, Akira S (2013). Autophagy in infection, inflammation and immunity. Nat Rev Immunol.

[24] Mizushima N, Yoshimori T (2007). How to interpret LC3 immunoblotting. Autophagy.

[25] Fortunato F, Burgers H, Bergmann F, Rieger P, Buchler MW, Kroemer G (2009). Impaired autolysosome formation correlates with Lamp-2 depletion: role of apoptosis, autophagy, and necrosis in pancreatitis. Gastroenterology.

[26] Dikic I (2017). Proteasomal and autophagic degradation systems. Annu Rev Biochem.

[27] Saitoh T, Fujita N, Jang MH, Uematsu S, Yang BG, Satoh T (2008). Loss of the autophagy protein Atg16L1 enhances endotoxin-induced IL-1beta production. Nature.

[28] Chung KW, Kim KM, Choi YJ, An HJ, Lee B, Kim DH (2017). The critical role played by endotoxin-induced liver autophagy in the maintenance of lipid metabolism during sepsis. Autophagy.

[29] Zhao H, Chen H, Xiaoyin M, Yang G, Hu Y, Xie K (2019). Autophagy activation improves lung injury and inflammation in sepsis. Inflammation.

[30] Galluzzi L, Green DR (2019). Autophagy-independent functions of the autophagy machinery. Cell.

[31] Mauvezin C, Neufeld TP (2015). Bafilomycin A1 disrupts autophagic flux by inhibiting both V-ATPase-dependent acidification and Ca-P60A/SERCA-dependent autophagosome-lysosome fusion. Autophagy.

[32] Mo Y, Lou Y, Zhang A, Zhang J, Zhu C, Zheng B (2018). PICK1 deficiency induces autophagy dysfunction via lysosomal impairment and amplifies sepsis-induced acute lung injury. Mediators Inflamm.

[33] Eskelinen EL (2006). Roles of LAMP-1 and LAMP-2 in lysosome biogenesis and autophagy. Mol Aspects Med.

[34] Colacurcio DJ, Nixon RA (2016). Disorders of lysosomal acidification-the emerging role of v-ATPase in aging and neurodegenerative disease. Ageing Res Rev.

[35] Chauhan S, Mandell MA, Deretic V (2015). IRGM governs the core autophagy machinery to conduct antimicrobial defense. Mol Cell.

[36] Dang TT, Kim MJ, Lee YY, Le HT, Kim KH, Nam S (2023). Phosphorylation of EIF2S1 (eukaryotic translation initiation factor 2 subunit alpha) is indispensable for nuclear translocation of TFEB and TFE3 during ER stress. Autophagy.

